# Simulated surgical-type cerebral biopsies from post-mortem brains allows accurate neuropathological diagnoses in the majority of neurodegenerative disease groups

**DOI:** 10.1186/2051-5960-1-53

**Published:** 2013-08-19

**Authors:** Andrew King, Satomi Maekawa, Istvan Bodi, Claire Troakes, Olimpia Curran, Keyoumars Ashkan, Safa Al-Sarraj

**Affiliations:** 1 Department of Clinical Neuropathology, Academic Neuroscience Building, Kings College Hospital, SE5 9RS Denmark Hill, London, England; 2 MRC London Neurodegenerative Diseases Brain Bank, Institute of Psychiatry, King’s College, London, UK; 3 Department of Clinical Neuroscience, King’s Health Partners Centre for Neurodegeneration Research, Institute of Psychiatry, King’s College, London, UK; 4 Department of Neurosurgery, King’s College Hospital, London, UK

**Keywords:** Neurodegeneration, Biopsy, Dementia

## Abstract

**Background:**

In theory, cerebral biopsies could provide the diagnosis in a significant proportion of patients with neurodegenerative diseases, however, there are considerable ethical barriers. Previous series of cerebral biopsies have shown variable diagnostic accuracy but have understandably suffered because of lack of post-mortem tissue with which to compare the diagnosis. To determine the accuracy of such biopsies in neurodegenerative disease we took small biopsy-sized samples of predominantly fresh post-mortem brain tissue from frontal and temporal lobes in 62 cases. These were processed as for a biopsy and stained for H&E, p62, tau, Aβ, α-synuclein, and TDP-43. The sections were assessed blind by 3 neuropathologists and the results compared with the final post-mortem diagnosis.

**Results:**

The agreement and sensitivity in most cases was good especially: controls; Alzheimer’s disease (AD); multiple system atrophy (MSA); frontotemporal lobar degeneration with TDP-43 positive inclusions and/or motor neurone disease (FTLD-TDP/MND); Huntington’s disease (HD); corticobasal degeneration (CBD) / microtubular associated protein tau mutation cases with CBD-like features (CBD/MAPT); and combined AD- Dementia with Lewy Bodies (AD-DLB) where the sensitivity on assessing both brain regions varied between 75-100%. There was poor sensitivity for progressive supranuclear palsy (PSP) and amyotrophic lateral sclerosis (ALS) (both 0%), but moderate sensitivity for pure DLB (60%). The temporal lobe assessment was marginally more accurate than the frontal lobe but these were only slightly worse than both combined.

**Conclusions:**

The study shows that with certain caveats the cerebral biopsy in life should be a viable method of accurately diagnosing many neurodegenerative diseases.

## Background

Despite significant advances in neuroradiological techniques and the emergence of possible biomarkers [1,2] the gold standard for diagnosis of neurodegenerative diseases remains the histological examination of post mortem brain tissue on the background of clinical evaluation. As new targeted therapies emerge the accurate diagnosis of neurodegenerative diseases in life may well become even more important. In theory, definitive diagnosis of several neurodegenerative diseases in life can be made on a surgically removed brain biopsy. There are, however, inherent risks associated with brain biopsies including seizures, bleeding and infection [3-5]. Whilst these risks are often deemed acceptable when attempting to diagnose potentially treatable severe conditions such as brain tumours, ethically there is much greater difficulty in justifying biopsies in non-tumour conditions where few treatment options exist. For this reason brain biopsies for potential neurodegenerative diseases have often been confined to cases where there has been a need to rule out some other possible treatable conditions such as infection or vasculitis. Because of this, such biopsies have tended to be done in younger to middle-aged patients rather than in those more prone to neurodegenerative disease [5]. There have been relatively few studies of the efficacy of brain biopsy in non-tumour conditions, especially those concentrating on the diagnosis of neurodegenerative disease [3-8]. By necessity the correct diagnosis in the majority of cases had to be evaluated by retrospective analysis of clinical features and scans with only a minority being able to be compared with a later post-mortem examination of the brain [5]. Because of these limitations up to 37% of the biopsies were considered non diagnostic, often the only features being non specific gliosis [5,7]. Venneti et al., however, using a simulated brain biopsy technique on partially-covered post-mortem brain slides attempted to determine the accuracy of biopsy interpretation by comparing the results with the final autopsy diagnosis [9]. They achieved relatively good sensitivity for many diseases when assessing frontal lobe only, but when 4 brain regions were assessed the accuracy increased considerably. However, they conceded that at least one of the four regions (basal ganglia) would probably not be appropriate for a routine biopsy procedure, and the analysis suffered a little on being retrospective in nature. The aim of this current study was therefore again to assess the accuracy of a simulated brain biopsy, but on this occasion by using fresh post-mortem brain tissue. The technique would allow sampling of the same potential biopsy sites as those targeted in life by neurosurgeons, whilst at the same time allowing good comparison with the final neuropathological diagnosis on the whole fixed brain.

## Methods

Formalin fixed paraffin embedded tissue from a total of 62 cases were obtained from the Medical Research Council (MRC) London Neurodegenerative Diseases Brain Bank, Institute of Psychiatry, King’s College London, London, UK. All subjects gave informed consent for tissue donation using procedures approved by the local Research Ethics Committee.

Following our neurosurgeon’s (KA) instruction, biopsy like samples of 1 cm^3^ cortical brain tissue from dorsolateral/anterior prefrontal (Brodmann area 9/10) and middle temporal cortex (Brodmann area 21) were dissected from 46 consecutive brain bank cases received between October 2011 and May 2012. The protocol for the MRC Brain Bank involves bisecting the brain prior to fixing one half and slicing and freezing the other, and the biopsy samples were taken from the half to be fixed. The tissue blocks were immediately fixed in 10% buffered formalin for one week and processed into paraffin blocks. In order to provide a greater balance and range of neurodegenerative diseases, one of the authors (CT) who was not involved in the histological assessment used fixed archival brains and provided biopsy sized samples from the same brain regions from 16 confirmed diagnoses of different neurological diseases and controls. These details remained blind to the histological evaluators.

The fixation times for those 16 cases ranged from 2-132 months. Those cases that were added in this fashion were Dementia with Lewy Bodies neocortical/limbic stages (DLB) [10] -2 cases; Corticobasal degeneration (CBD) or microtubular associated protein tau (MAPT) mutation-with CBD features (CBD/MAPT)-4 cases (because even after post-mortem evaluation of the brain, the cases of MAPT mutations seen here were indistinguishable from CBD and without genetic analysis it was considered that for this particular study they should be considered as one group); Multiple system atrophy (MSA) -2 cases; Progressive supranuclear palsy (PSP)-2 cases; Pure Alzheimer’s Disease (AD) (Modified Braak/ Brain Net Europe (BNE) Stage IV-VI) [11] -2 cases; Huntington’s Disease (HD)- 2 cases; Controls (no history of cognitive decline or movement disorder, maximum modified Braak (BNE) AD stage of II) [11] - 2 cases.

The autopsy confirmed cases therefore overall were as follows:

Controls - 9 cases; pure AD (Modified Braak (BNE) Stage IV-VI [11] - 15 cases; PSP -3 cases; CBD/MAPT- 7 cases; neocortical/limbic stages of DLB [10] - 5 cases; MSA - 3 cases; HD-6 cases; frontotemporal lobar degeneration with TDP-43 positive inclusions with or without motor neurone disease (FTLD-TDP/FTLD-MND)-6 cases; metabolic diseases (Batten’s Disease)-1 case; pure cerebrovascular disease -1 case; Combined AD (Modified Braak (BNE) Stage IV-VI) [11] and DLB (neocortical/limbic stage) [10] - 4 cases; Amyotrophic Lateral Sclerosis (ALS) (no extramotor inclusions) - 2 cases. The details of the cases are illustrated in Table 1.

**Table 1:** Showing the number and range of cases from which simulated brain biopsies were taken

**Cases**	**Number in group**	**Relevant stages**	**Comments**
Control	9	AD (BNE/Braak) 0-II [11]	
AD	15	AD (BNE/Braak) IV-VI	2 cases mild cortical TDP-43
PSP	3		
CBD	5 Total = 7		
MAPT (with CBD pathology)	2		
DLB	5	Limbic [10] - 3	
		Neocortical - 2	
MSA	3		
HD	6		
FTLD-TDP/FTLD-MND	6	FTLD-TDP -1	1 case C9orf72 mutation
		FTLD-MND -5	
Metabolic disease	1	Batten’s disease	CLN2 mutation
Cerebrovascular disease	1		Infarct and amyloid angiopathy
ALS	2		No extramotor inclusions
Combined AD and DLB	4	AD (BNE Braak) (V-VI)	1 case mild cortical TDP-43
		DLB Limbic -2	
		DLB Neocortical - 2	

**Figure 1:**
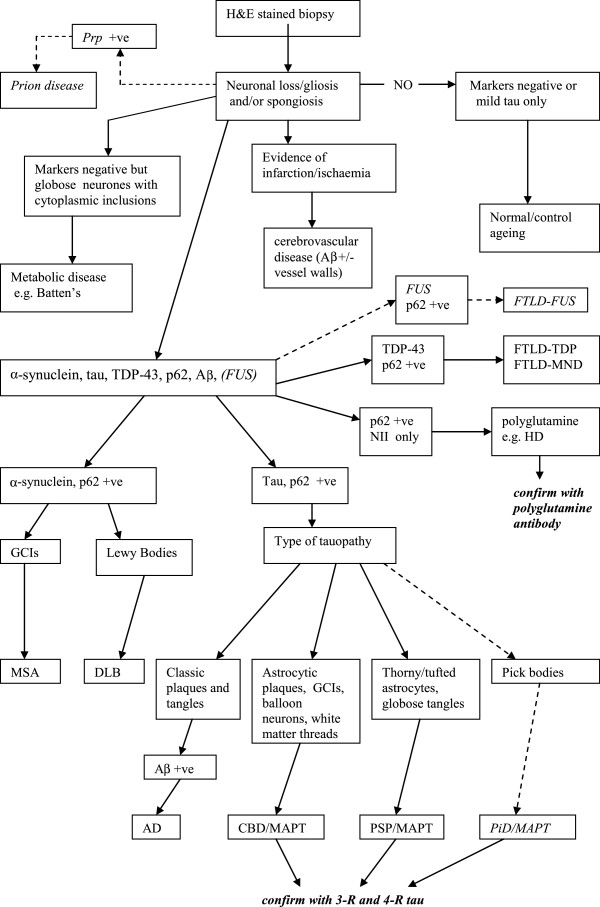
**Suggested diagnostic algorithm for cerebral biopsies in the diagnosis of neurodegenerative diseases based on the simulated biopsy technique.** The broken lines and italics indicates groups of diseases which were not actually assessed in this study.

Because of the relevant strict Health and Safety procedures in dealing with fresh tissue it was not considered possible to use prion disease cases in the evaluation [12]. All the blocks were cut at 7 μm thickness and stained with haematoxylin and eosin (H&E). Sections were also immunohistochemically stained with the mouse monoclonal antibody to p62 (1:100; BD Biosciences, Erembodegem, Belgium), phosphorylated tau (clone [AT-8]; 1:500; Autogen Bioclear UK Ltd, Wiltshire, UK), α-synuclein (clone [42/α synuclein]; 1:500; Novocastra Laboratories Ltd, Newcastle-upon-Tyne, UK), and Amyloid β (Aβ) (1:12000; Chemicon, Temecula, CA, USA), or the rabbit polyclonal antibody to phosphorylated TDP-43 (pS409/410-2; 1:1500); Cosmo Bio Ltd, Tokyo, Japan) using the Leica BONDMAX™ (Leica Biosystems, Wetzlar, Germany). Leica BONDMAX™ epitope retrieval sets was used for TDP-43 (ER1 for 30 minutes), p62 (ER2 for 20 minutes) and tau (ER1 for 20 minutes). For both α synuclein, and Aβ 80% formic acid pre-treatment (for 1 hour) was used. Nuclei were counterstained with Harris’ alum hematoxylin. All cases were examined independently by the three neuropathologists (SAS, AK, IB) blind to clinical information, final post-mortem diagnoses and overall range of final diagnoses. All immunostained sections were semi-quantitatively rated as having no (-), only very occasional (+/-), mild (+), moderate (++) or severe (+++) immunoreactivity. The frontal and temporal lobe biopsies were assessed both separately and combined by each assessor and then a diagnosis attempted on each block separately and then both combined. Briefly the characteristic histological features searched for were tau positive neuritic plaques, neuropil threads and neurofibrillary tangles and Aβ positive plaques in AD; α-synuclein and p62 positive Lewy bodies and Lewy Neurites in DLB; α-synuclein and p62 positive oligodendroglial cell inclusions in MSA; TDP-43 and p62 positive inclusions in FTLD-TDP/MND (and ALS) [13]; tau positive globose tangles and tufted astroctyes in PSP; tau positive, p62 positive astrocytic plaques and possible balloon cells in CBD/MAPT; Pick bodies in Pick’s Disease (PiD); p62 positive, TDP-43 negative neuronal intranuclear inclusions (NII) in HD; infarction, or severe ischaemic changes with vascular wall changes including possible amyloid angiopathy for cerebrovascular disease. An adapted associated algorithm for the diagnostic approach is shown in Figure 1[7,9,14,15]. The final gold standard autopsy diagnoses were made using the consensus criteria available for DLB [10], and the BNE staging for AD [11]. In other cases agreement was reached by the 3 pathologists after examining the autopsy slides, with access to relevant clinical and genetic details but totally blind to the results of the biopsy assessments.

Statistical evaluation of the 62 cases (against the 12 categories of disease stated above) was made using an online statistical package (http://www.statstodo.com). Statistical significance was set at the 0.05 level. Agreement between the frontal and temporal lobe assessments (both separately and combined) and the gold standard autopsy diagnosis was assessed with the kappa coefficient. Point estimates and 95% confidence intervals (CI) were obtained for the kappa coefficient. Kappa values were interpreted using proposals of Landis and Koch [16]; kappa values below 0 indicate “poor” agreement, 0-0.20 “slight” agreement, 0.21-0.40 “fair” agreement, 0.41-0.60 “moderate” agreement, 0.61-0.80 “substantial” agreement, and 0.81-1.00 “almost perfect” agreement. Within each group overall sensitivity was calculated as the probability of a positive test given that the patient had the disease, and the specificity overall as the probability of a negative test given that the patient did not have the disease. For this purpose all 3 raters had to be in agreement with the particular final diagnosis to indicate a positive test.

## Results

**Figure 2:**
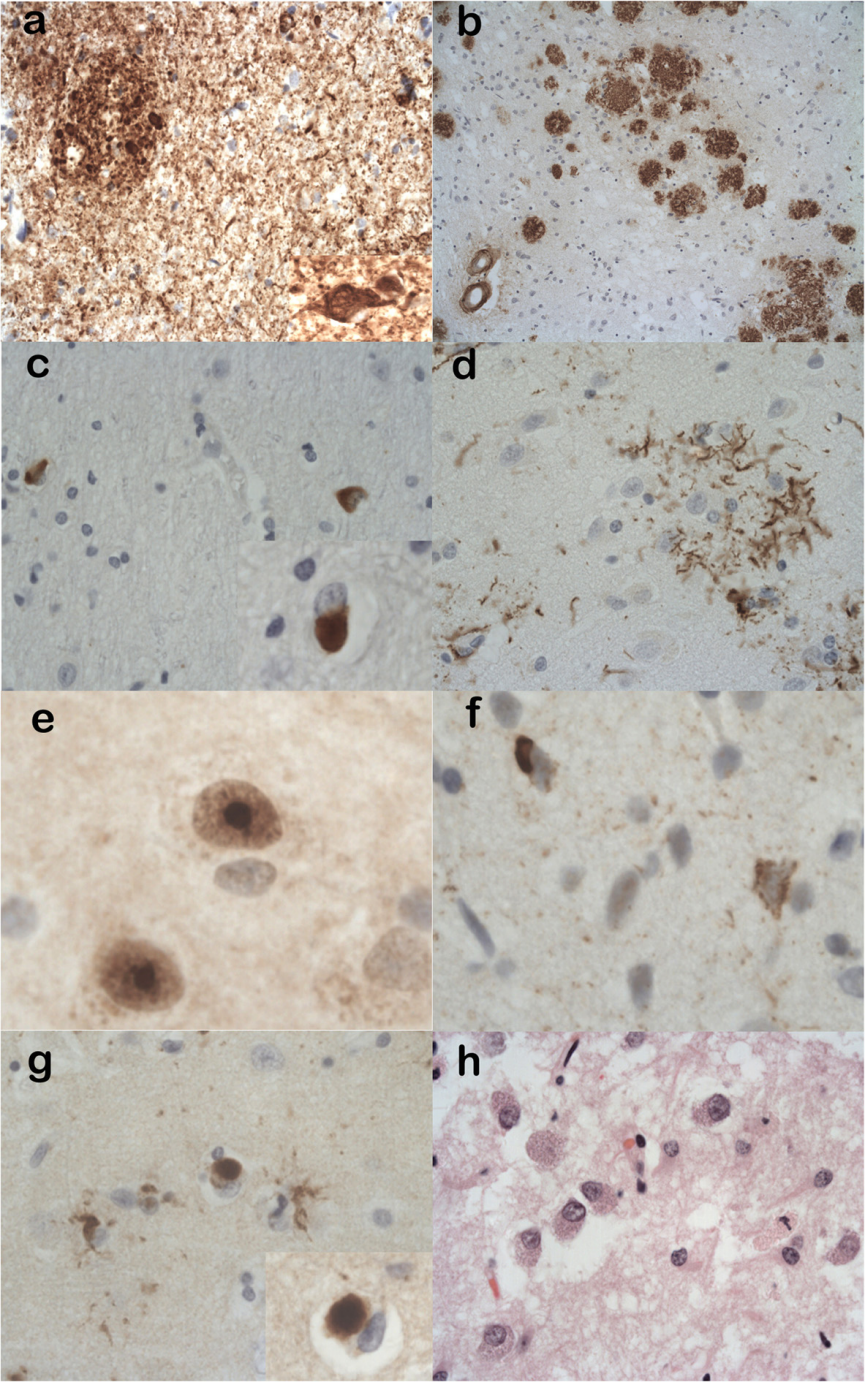
**Histological features from simulated biopsies of neurodegenerative diseases diagnosed in the study. (a)** Alzheimer’s disease illustrating the high density of tau positive neuropil threads, neuritic plaques and a neurofibrillary tangle (inset) (anti-tau). **(b)** Alzheimer’s disease revealing Aβ positive plaques in the cortex and evidence of amyloid angiopathy (anti-Aβ). **(c)** Multiple system atrophy (MSA) showing numerous p62 positive and α-synuclein positive (inset) oligodendroglial cytoplasmic inclusions in cerebral white matter. **(d)** Corticobasal degeneration showing a tau positive astrocytic plaque in the cortex (anti-tau). **(e)** Huntington’s disease revealing p62 positive intranuclear inclusions within neurones (anti-p62). **(f)** Frontotemporal lobar degeneration with TDP-43 positive inclusions (FTLD-TDP) showing TDP-43 positive neuronal cytoplasmic inclusions in the neocortex (anti-TDP-43). **(g)** Dementia with Lewy bodies illustrating α-synuclein positive Lewy bodies in the neocortex and a Lewy body at higher magnification (inset) (anti-α-synuclein). **(h)** Batten’s disease (neuronal ceroid lipfuscinosis type 2) showing enlarged neurones with excess granular cytoplasmic material (H&E). Main pictures **(a)**, **(b)** show ×20 magnifications; **(c)**, **(d) (g)** show ×40 magnifications, **(f) (h)** and inset for **(a)** show ×60 magnifications. **(e)** and inset for **(c)** show ×120 magnifications and inset for **(g)** shows × 100 magnification.

The overall results of the assessments are summarised in Table 2. In addition, the histological features for a range of the neurodegenerative diseases diagnosed are illustrated in Figure 2.

**Table 2:** Illustrating the diagnostic accuracy of the assessors for particular disease groups when examining the simulated biopsies from the frontal lobe, the temporal lobe and combined

**Cases**	**No.**	**Correct diagnosis**	**Comments**
		**C-combined, F-Frontal, T-Temporal**	
Control	9	C-9 (100%), F-100%, T-100%	
AD	15	C-15 (100%), F-100%, T-100%	
PSP	3	C-0 (0%), F-0%, T-0%	1 assessor detected*
CBD/MAPT	7	C- 7 (100%), F-100%, T-100%	
DLB	5	C- 3 (60%), F-60%, T-40%	
MSA	3	C- 3 (100%), F-100%, T-100%	
HD	6	C- 5 (83%), F-83%, T-83%	1 assessor missed†
FTLD-TDP/FTLD-MND	6	C- 5 (83%), F-67%, T-83%	1-2 assessor(s) missed #
Metabolic disease	1	C-1 (100%), F-100%, T-100%	
Cerebrovascular disease	1	C-1 (100%), F-100%, T-100%	
ALS	2	C- 0 (0%), F-0%, T-0%	
Combined AD and DLB	4	C-3 (75%), F-75%, T-75%	1 assessor missed in limbic DLB case~

*- 1 assessor diagnosed PSP in one case only.

†- 1 assessor missed diagnosis in one case.

# - 1 assessor missed diagnosis in one case, 1 assessor missed another diagnosis in another case when examining frontal lobe.

~ - 1 assessor missed diagnosis in one combined AD-DLB case when DLB component was of limbic stage.

Overall there was very good agreement with the gold standard autopsy diagnosis (frontal lobe alone kappa = 0.84, 95% CI = 0.80 - 0.88, p<0.001, temporal lobe alone kappa = 0.86, 95% CI= 0.82 - 0.90, p<0.001 combined kappa = 0.87, 95% CI= 0.83 - 0.91, p<0.001). Therefore assessing the temporal lobe alone was marginally more accurate than assessing the frontal lobe alone but both yielded excellent results and this accuracy increased only slightly when combining the assessment of both biopsy sites.

The overall sensitivity for combined sites was 85%, compared to 81% for the frontal lobe alone, and 84% for the temporal lobe alone. The overall specificity for combined sites was 83% compared to 79% for the frontal lobe alone, and 82% for the temporal lobe alone.

In occasional cases the neuropathologists were in agreement with each other but nonetheless were unable to diagnose the correct disease. For example in 2 of the 3 cases of PSP all the assessors diagnosed “controls” (the other case of PSP was only detected by 1 assessor) (0% sensitivity). Again in both the ALS cases (0% sensitivity) as well as 2 of the 5 DLB (60% sensitivity) cases there was consensus between the assessors but with an an incorrect diagnosis. In contrast, on the combined assessment there was complete agreement (100% sensitivity) for the cases of pure AD, CBD/MAPT, MSA, cerebrovascular disease, metabolic disease, and controls. There was a single rater discrepancy in only 1 of the 6 cases of FTLD-TDP/MND (83% sensitivity), 1 of the 6 cases of HD (83% sensitivity) and 1 of the 4 cases of combined AD-DLB (75% sensitivity). In addition, occasional incidental TDP-43 inclusions were noted by all raters in 2 of the cases of AD and 1 case of combined AD-DLB. The temporal lobe biopsy (83% sensitivity) appeared slightly better at detecting TDP-43 inclusions in cases of FTLD-TDP/MND than the frontal lobe biopsy (67% sensitivity) but the frontal lobe biopsy (60% sensitivity) detected Lewy Bodies in one case of DLB, which was not present on the temporal lobe biopsy (40% sensitivity). In many cases of AD the tau pathology was more abundant in the temporal lobe biopsy than the frontal lobe biopsy, but this did not appear to affect the final diagnosis either when the biopsies were analysed separately or together. Because of excess p62 pathology one rater correctly suggested a c9orf72 repeat expansion in one case of FTLD-MND [17,18]. The suggested limitations and usefulness of the biopsy technique depicted here is illustrated in Table 3.

**Table 3:** Illustrating the likely usefulness and limitations of cerebral biopsies in the diagnosis of neurodegenerative diseases

**Disease**	**Likely usefulness of cerebral biopsy**	**Frontal or temporal site preferred (F/T)**	**Stains recommend in first instance**	**Secondary stains confirmatory**	**Secondary stains to exclude other diagnoses**	**Other information required**	**Comments**
AD (IV-VI)	High	F or T	HE, tau, p62, Aβ		TDP-43, α-syn	History, scans	
PSP	Low	N/A	HE, p62, tau	4-R tau,	Aβ, TDP-43, α-syn	History, scans, genetics	Negative biopsy does not exclude. Rule out MAPT mutation FTD and/or other parkinsonian diseases
				3-R tau			
CBD	High	F or T	HE, p62, tau	4-R tau,	Aβ, TDP-43, α-syn	History, scans, genetics	Rule out MAPT mutation
				3-R tau			
MAPT mutation	Likely to depend on mutation	N/A	HE, p62, tau	4-R tau,	Aβ, TDP-43, α-syn	History, scans, genetics	Genetics essential for diagnosis
				3-R tau			
DLB	High- neocortical, Moderate-Low - limbic	F >T	HE, p62, α-syn,		Aβ, tau, TDP-43	History, scans	Often seen with AD pathology
MSA	High	F or T	HE, p62, α-syn,		tau	History, scans	White matter essential in the biopsy
HD	High	F or T	HE, p62	polyglutamine	TDP-43, FUS	History, scans, genetics	Genetics essential for diagnosis
FTLD-TDP/ FTLD-MND	High	T > F	HE, p62, TDP-43		Aβ, tau, FUS	History, scans, genetics	History to tell FTLD-MND from FTLD-TDP
ALS	Very low-nil	N/A	HE, p62, TDP-43, FUS			History, scans genetics	Biopsy-Very unlikely to help
Metabolic disease	Likely to depend on disease	N/A	HE, PAS, LFB/N		p62, tau	History, scans genetics	
CVD	Depends what type: infarct-high AA-high Binswanger’s-low	N/A	HE, Aβ, CR		p62, tau, LFB/N	History, scans	Leptomeninges needed for AA, Binswanger’s likely to be missed without deep white matter
*Prion Disease*	*Moderate-High*	*unknown*	*HE, GFAP, PrP*		*Aβ, tau, TDP-43, α-syn*	*History, scans genetics*	*Negative biopsy does not rule out diagnosis*
*FTLD-FUS*	*unknown*	*unknown*	*HE, p62, FUS*		*Aβ, tau, TDP-43, α-syn*	*History, scans genetics*	
*PiD*	*High*	*unknown*	*HE, p62, tau*	*4-R tau, 3-R tau*	*TDP-43, Aβ, α-syn*	*History, scans genetics*	*Rule out MAPT mutation*
*PD*	*Very low-nil*	*N/A*	*HE, p62 α-syn*		*tau*	*History, scans genetics*	*Biopsy could rule out other causes of parkinsonism*

Those cases in italics were not included in this study but features have been included and estimated from the following references [3,5,7,9]. *AA* amyloid angiopathy, *CVD* cerebrovascular disease, *CR* congo red, *F* frontal lobe, *FTD* frontotemporal dementia, *GFAP* glial fibrillary acidic protein, *LFB/N* Luxol fast blue/Nissl, *N/A* details not applicable, *PAS* Periodic acid Schiff, *PiD* Pick’s disease, *PD* Parkinson’s disease, *PrP* prion protein, *α-syn* α-synuclein, *T* temporal lobe.

> - estimated to be more slightly more sensitive site than.

## Discussion

The potential risks of surgical biopsy are not insignificant with an estimated complication rate of usually between 5-11% but occasionally reaching 21%. These complications include haemorrhage, neurological deficit, infection, anaesthetic complications, eplilepsy and death [3-5,7]. Thus for tumours where treatment options are available biopsies are commonly performed. When considering such biopsies for non-tumour conditions there is obviously an ethical dimension to take into account. For this reason such biopsies have tended to be confined to potentially treatable conditions such as suspected vasculitis or infection rather than dementias. They have also tended to be performed in younger patients than those commonly affected with neurodegenerative diseases [7]. Nevertheless, biopsies in suspected neurodegenerative diseases have occasionally been performed in an attempt to rule out an alternative treatable cause of the cognitive decline or movement disorder [3-8,19]. Whilst some clinicians consider the procedure in this context a necessary evil even if it only provides information to a patient’s relatives, and allows the cessation of non necessary treatment, others consider it to be highly dubious ethically, of little therapeutic use and indeed they have raised concerns over obtaining informed consent for the procedure [3,20,21]. If particular disease specific therapies emerge prior to the validation of associated disease biomarkers the use of cerebral biopsies may become an important tool in the diagnosis of neurodegenerative diseases in life. So far there is little evidence as to the accuracy of the procedure in such diseases. Those studies that have occurred have been mainly biopsy series with little or no autopsy confirmation of the diagnoses. The accuracy in diagnoses in such series has necessarily involved retrospective analysis of scans, clinical parameters and subsequent disease course and (including all non-tumour conditions, not just neurodegenerative diseases) has varied between 53% and 77% [4-8,19]. There were a number of cases which gave a non specific diagnosis. It was interesting that the follow up study at one centre showed an improvement in diagnosis from 57 to 74% over a 5 year period, perhaps reflecting greater range of specific antibodies available and greater experience on the part of the pathologists [5,7]. However, it was also noted that the number of biopsies had decreased perhaps with the physicians concentrating on those most likely to give a definitive answer. Venneti et al. mindful of needing to provide a definitive answer when assessing the accuracy of cerebral biopsies in neurodegenerative diseases used partially covered post-mortem slides to simulate brain biopsies [9]. They achieved a very good sensitivity and inter-rater reliability for most cases, when assessing 4 brain regions and these remained high when examining frontal lobe only. The authors conceded that one of the regions (basal ganglia) would be less likely biopsied in life, also that there were some constraints when assessing in a retrospective manner such as this. Our study tried as much as possible to simulate a cerebral biopsy in life by taking small samples from the frontal and temporal regions and in most cases fixing and processing as one would a biopsy rather than autopsy blocks.

By using this simulated brain biopsy technique we examined 62 cases and compared the assessments with the final post-mortem diagnosis. With the majority of neurodegenerative diseases assessed we managed high accuracy using either the frontal or temporal lobe or both together. The pathologists were particularly accurate at detecting pure AD, CBD/MAPT, MSA, combined AD-DLB, FTLD-TDP/MND, HD, cerebrovascular disease, metabolic disease, and controls. They were, however, poor at detecting PSP and this agrees with the findings of Venneti et al. and probably reflects the relative paucity of tau expression in the neocortex and white matter when compared with brain stem, basal ganglia and cerebellar pathology [9]. Since 2 of the 3 cases of PSP were from the archival tissue (fixation times of 5 years and 2 years respectively) there is a possibility that this prolonged fixation time had deleterious effects on the expression of the tau antibody. Dwork et al. however, when assessing this phenomenon with the Alz-50 antibody (later seen to recognise a phosphorylated epitope of tau) showed that whilst this effect does occur, it is not often significant until 20 years have elapsed [22]. The assessors were also relatively poor at assessing pure DLB, but here the cases that were not detected were limbic stage, (both alone or combined with AD) and on analysis of the final post-mortem brain the α-synuclein pathology was not indeed present in the location of the simulated biopsy. Those cases missed by the assessors tended to be in the limbic stage according to the McKeith classification, in that at autopsy they had only occasional Lewy Bodies in the middle frontal and/or temporal neocortex but not the parietal neocortex [10]. The 2 pure neocortical stages of pure DLB were both diagnosed by all participants from both biopsy regions and the 2 neocortical stages of DLB when combined with AD were both also recognised. Not surprisingly, none of the participants could detect any pathology in the ALS cases since there was no extramotor pathology seen in the autopsy brains. What was particularly encouraging was the detection of MSA cases even with relatively scanty white matter and especially considering the fact that these cases were submitted from archival formalin wet tissue and α-synuclein staining is somewhat susceptible to prolonged fixation [23]. This would be a somewhat hypothetical problem since in a live biopsy scenario prolonged fixation would be unlikely. Furthermore, it was noted that the p62 was a sensitive marker for detecting the characteristic “cup and ball” oligodendroglial inclusions of MSA. Indeed p62 although non specific labels Lewy bodies, neurofibrillary tangles, glial inclusions of tauopathies and MSA, inclusions in FTLD-TDP/MND as well as the intranuclear inclusions of polyglutamine disorders such as HD [24-26]. In this study therefore p62 proved to be invaluable as a primary “screening” stain. It was also interesting to note that the staining protocol was sensitive enough to detect secondary mild cortical TDP-43 pathology in 2 cases of pure AD and one case of mixed AD-DLB. The significance of this TDP-43 pathology in association with other diseases is somewhat obscure although there is one study that does show such abnormal TDP-43 expression may correlate with older age and different clinical features [27]. In the context seen here it could theoretically modify any potential therapy or at least give a clue as to the overall disease progression. The detection of HD by p62 positive,TDP-43 negative neuronal intranuclear staining again is an interesting yet somewhat academic finding since aside from those patients with atypical clinical features the current availability of a genetic test for HD would render such a biopsy of little practical use.

One criticism that could be made of the study would be that prion diseases were excluded. The main reason for this is the Health and Safety aspects of dealing with such cases (especially fresh brain tissue). The difficulty in dealing with biopsies from prion disease cases has already been more extensively studied [7]. In any case the algorithm could be easily adapted to take into account of this particular possible diagnosis and as well as performing immunohistochemistry fresh tissue could be sent for analysis of the prion proten subtypes [28,29].

Again no PiD cases were present in our sample although judging from the results here they would expected to present little diagnostic difficulties on an adequately-sized biopsy. Venneti et al. comfirmed a high rate of detection in such cases [9]. Cases of frontotemporal lobar degeneration with fused in sarcoma (FUS) positive inclusions (FTLD-FUS) are rare and were not seen in our series. Potentially they could be important because they often present in younger or middle-aged patients. This should theoretically present little difficulty in that the neuronal cytoplasmic inclusions should be p62 positive, and TDP-43 negative and the addition of FUS immunohistochemistry to the repertoire should lead to the diagnosis [30,31]. When cerebral biopsies for non-tumour conditions are taken they are usually from the non-dominant hemisphere and in our cases it was considered more important to assess the simulated biopsies from the same side as the fixed cerebral hemisphere, and since the protocol for the MRC brain bank requires alternating hemispheres for fixation this could be seen as a potential deviation when compared with in vivo biopsies. This however, would be unlikely to represent a problem apart from the occasional cases of CBD and/or FTLD-TDP where there is marked asymmetry in degeneration and pathology (not seen in this series). The vast majority of the specimens contained leptomeninges by which to judge the presence and severity of amyloid angiopathy, but there was seldom sufficient white matter by which to judge diffuse white matter cerebrovascular disease such as “Binswanger’s disease”.

The frontal lobe is the most widely biopsied site and whilst the results here show slightly worse accuracy when compared with the temporal lobe (either alone or combined) it still appears to represent a sensitive area to assess apart from in PSP and the limbic stages of DLB. It should be remembered that these biopsies were assessed blind to clinical and radiological details. Therefore, in a real life situation where these additional assessments and results would be available, the accuracy may well be higher, and the diagnostic algorithm could be adjusted such that those cases with either a high index of suspicion for PSP or limbic DLB could either not be biopsied at all (instead relying on other criteria) or else a negative biopsy would not be taken to absolutely exclude the diagnosis. Similarly, if FTLD-TDP/MND was in the differential diagnosis there could be consideration as to whether a temporal biopsy be taken so as to increase the chance of a positive diagnosis. In addition, as illustrated in Figure 1 the accuracy of the biopsy technique could be further enhanced by the use of specific stains such as 4-repeat and 3-repeat tau in the tauopathies and polyglutamine antibodies in HD. The purpose of attempting to establish biomarkers for various neurodegenerative disease is so that there can be detection of a particular disease by a relatively non invasive technique such that the prognosis can be attempted and hopefully in the future therapeutic options can be considered [32]. Ideally the biomarker should detect the condition early so that disease course can be prevented or at least slowed down. One could argue that the simulated biopsy technique seen here only allows detection of the established (or end-stage) pathology and therefore is likely to be of little practical use when possible therapies are being considered. It may indeed be that that the actual taking of the biopsy at the earlier stage could just reveal non specific gliosis and this may explain the significant proportion of such diagnoses seen in biopsy series [5,7,8]. Even if this were the case, phase II studies in clinical trials still involve testing therapies in established disease groups and the brain biopsy technique may be particularly useful in this situation.

## Conclusions

Therefore we have shown in this study that with certain caveats the simulated brain biopsy technique can accurately detect the majority of neurodegenerative diseases. The brain biopsy may become a useful diagnostic tool when considering treatment of these diseases with potential targeted therapies.

## Competing interest

The authors declare they have no competing interest.

## Authors’ contributions

AK, SM, SAS, CT, IB, OC, KA designed the study. AK, IB, SAS made the assessments. CT added additional cases. AK and SM drafted the manuscript. All authors contributed to the manuscript and approved the final manuscript.

## References

[B1] Hu WT Chen-Plotkin A Arnold SE Biomarker discovery for Alzheimer's disease, frontotemporal lobar degeneration, and Parkinson's disease Acta Neuropathol 2010 1 385 399 10.1007/s00401-010-0723-9 20652578 PMC2982700

[B2] Wu Y Le W Jankovic J Preclinical biomarkers of Parkinson disease Arch Neurol 2011 1 22 30 10.1001/archneurol.2010.321 21220674

[B3] Hulette CM Earl NL Crain BJ Evaluation of cerebral biopsies for the diagnosis of dementia Arch Neurol 1992 1 28 31 10.1001/archneur.1992.00530250032011 1728259

[B4] Pulhorn H Quigley DG Bosma JJ Impact of brain biopsy on the management of patients with nonneoplastic undiagnosed neurological disorders Neurosurgery 2008 1 833 837 10.1227/01.neu.0000318168.97966.17 18496189

[B5] Warren JD Schott JM Fox NC Brain biopsy in dementia Brain 2005 1 2016 2025 10.1093/brain/awh543 15901648

[B6] Josephson SA Papanastassiou AM Berger MS The diagnostic utility of brain biopsy procedures in patients with rapidly deteriorating neurological conditions or dementia J Neurosurg 2007 1 72 75 10.3171/jns.2007.106.1.72 17236490

[B7] Schott JM Reiniger L Thom M Brain biopsy in dementia: clinical indications and diagnostic approach Acta Neuropathol 2010 1 327 341 10.1007/s00401-010-0721-y 20640903

[B8] Wong SH Jenkinson MD Faragher B Brain biopsy in the management of neurology patients Eur Neurol 2010 1 42 45 10.1159/000315032 20606447

[B9] Venneti S Robinson JL Roy S Simulated brain biopsy for diagnosing neurodegeneration using autopsy-confirmed cases Acta Neuropathol 2011 1 737 745 10.1007/s00401-011-0880-5 21959586 PMC3575084

[B10] McKeith IG Dickson DW Lowe J Diagnosis and management of dementia with Lewy bodies: third report of the DLB consortium Neurology 2005 1 1863 1872 10.1212/01.wnl.0000187889.17253.b1 16237129

[B11] Alafuzoff I Arzberger T Al-Sarraj S Staging of neurofibrillary pathology in Alzheimer's disease: a study of the BrainNet Europe Consortium Brain Pathol 2008 1 484 496 18371174 10.1111/j.1750-3639.2008.00147.x PMC2659377

[B12] Ironside JW Bell JE The 'high-risk' neuropathological autopsy in AIDS and Creutzfeldt-Jakob disease: principles and practice Neuropathol Appl Neurobiol 1996 1 388 393 10.1111/j.1365-2990.1996.tb00908.x 8930948

[B13] Cairns NJ Bigio EH Mackenzie IR Neuropathologic diagnostic and nosologic criteria for frontotemporal lobar degeneration: consensus of the consortium for frontotemporal lobar degeneration Acta Neuropathol 2007 1 5 22 10.1007/s00401-007-0237-2 17579875 PMC2827877

[B14] Kleinschmidt-De Masters BK Prayson RA An algorithmic approach to the brain biopsy--part I Arch Pathol Lab Med 2006 1 1630 1638 17076524 10.5858/2006-130-1630-AAATTB

[B15] Prayson RA Kleinschmidt-DeMasters BK An algorithmic approach to the brain biopsy–part II Arch Pathol Lab Med 2006 1 1639 1648 17076525 10.5858/2006-130-1639-AAATTB

[B16] Landis JR Koch GG The measurement of observer agreement for categorical data Biometrics 1977 1 159 174 10.2307/2529310 843571

[B17] Al-Sarraj S King A Troakes C p62 positive, TDP-43 negative, neuronal cytoplasmic and intranuclear inclusions in the cerebellum and hippocampus define the pathology of C9orf72-linked FTLD and MND/ALS Acta Neuropathol 2011 1 691 702 10.1007/s00401-011-0911-2 22101323

[B18] Murray ME Dejesus-Hernandez M Rutherford NJ Clinical and neuropathologic heterogeneity of c9FTD/ALS associated with hexanucleotide repeat expansion in C9ORF72 Acta Neuropathol 2011 1 673 690 10.1007/s00401-011-0907-y 22083254 PMC3277860

[B19] Rice CM Gilkes CE Teare E Brain biopsy in cryptogenic neurological disease Br J Neurosurg 2011 1 614 620 10.3109/02688697.2010.551677 21501048

[B20] Hauw JJ Duyckaerts C Dementia, the fate of brain? Neuropathological point of view C R Biol 2002 1 655 664 10.1016/S1631-0691(02)01479-8 12365416

[B21] Javedan SP Tamargo RJ Diagnostic yield of brain biopsy in neurodegenerative disorders Neurosurgery 1997 1 823 828 10.1097/00006123-199710000-00011 9316043

[B22] Dwork AJ Liu D Kaufman MA Prohovnik I Archival, formalin-fixed tissue: its use in the study of Alzheimer's type changes Clin Neuropathol 1998 1 45 49 9496540

[B23] Pikkarainen M Martikainen P Alafuzoff I The effect of prolonged fixation time on immunohistochemical staining of common neurodegenerative disease markers J Neuropathol Exp Neurol 2010 1 40 52 10.1097/NEN.0b013e3181c6c13d 20010304

[B24] Furukawa Y Iseki E Hino H Ubiquitin and ubiquitin-related proteins in the brains of patients with atypical Pick's disease without Pick bodies and dementia with motor neuron disease Neuropathology 2004 1 306 314 10.1111/j.1440-1789.2004.00572.x 15641590

[B25] Kuusisto E Kauppinen T Alafuzoff I Use of p62/SQSTM1 antibodies for neuropathological diagnosis Neuropathol Appl Neurobiol 2008 1 169 180 10.1111/j.1365-2990.2007.00884.x 17961133

[B26] Kuusisto E Parkkinen L Alafuzoff I Morphogenesis of Lewy bodies: dissimilar incorporation of alpha-synuclein, ubiquitin, and p62 J Neuropathol Exp Neurol 2003 1 1241 1253 14692700 10.1093/jnen/62.12.1241

[B27] Davidson YS Raby S Foulds PG TDP-43 pathological changes in early onset familial and sporadic Alzheimer's disease, late onset Alzheimer's disease and down's syndrome: association with age, hippocampal sclerosis and clinical phenotype Acta Neuropathol 2011 1 703 713 10.1007/s00401-011-0879-y 21968532

[B28] Parchi P Castellani R Capellari S Molecular basis of phenotypic variability in sporadic Creutzfeldt-Jakob disease Ann Neurol 1996 1 767 778 10.1002/ana.410390613 8651649

[B29] Parchi P Giese A Capellari S Classification of sporadic Creutzfeldt-Jakob disease based on molecular and phenotypic analysis of 300 subjects Ann Neurol 1999 1 224 233 10.1002/1531-8249(199908)46:2<224::AID-ANA12>3.0.CO;2-W 10443888

[B30] Lashley T Rohrer JD Bandopadhyay R A comparative clinical, pathological, biochemical and genetic study of fused in sarcoma proteinopathies Brain 2011 1 2548 2564 10.1093/brain/awr160 21752791 PMC3170529

[B31] Neumann M Rademakers R Roeber S A new subtype of frontotemporal lobar degeneration with FUS pathology Brain 2009 1 2922 2931 10.1093/brain/awp214 19674978 PMC2768659

[B32] Wagner JA Biomarkers: principles, policies, and practice Clin Pharmacol Ther 2009 1 3 7 10.1038/clpt.2009.77 19536113

